# Introducing Nitramide Group into High Energy Density Material Molecule Leads to Enhanced Performance

**DOI:** 10.1021/jacsau.5c00411

**Published:** 2025-07-02

**Authors:** Yi Wang, Shichao Liu, Wei Le, Sergey V. Zybin, Wanjun Zhao, Fenglei Huang, William A. Goddard III, Dezhou Guo

**Affiliations:** † State Key Laboratory of Explosion Science and Safety Protection, 47833 Beijing Institute of Technology, Beijing 10081, China; ‡ Materials and Process Simulation Center, 6469 California Institute of Technology, Pasadena, California 91125, United States

**Keywords:** BCHMX, USPEX, Chapman−Jouguet state, Oxygen Balance, Energetic Performance

## Abstract

Although it has been verified by many experimental studies that the design of introducing nitrogen-rich groups into current molecular backbones is a practical method to increase the detonation properties, there is no clear understanding of how energetic explosophores would affect the energy storage density and energy release degree of high energy density materials (HEDMs). The BCHMX (cis-1,3,4,6-tetranitrooctahydroimidazo-[4,5-*d*]­imidazole) molecule was designed based on the HMX (1,3,5,7-tetranitro-1,3,5,7-tetrazocane) molecule by introducing intramolecular carbon–carbon linkages, which provides an excellent spot to introduce a nitramide group. Thus, we designed the BCHMX-ENO (2,4,6,8,9-pentanitro-2,4,6,8,9-pentaazabicyclo[3.3.1]­nonane) molecule. To examine its properties, we first employed evolutionary algorithms USPEX to predict the crystal structure of BCHMX-ENO. Then, we applied QM-MD (quantum mechanics molecular dynamics) simulations to examine the initial thermal decomposition reactions and used a combination of RxMD (reactive molecular dynamics with ReaxFF force field) and QM-MD simulations to predict the detonation performance of BCHMX and BCHMX-ENO. We found that nitramide group influences initial reaction steps by affecting the molecular spatial distribution, bond length, and atom distance. We predicted that BCHMX-ENO shows improved detonation properties with 7.40% higher Chapman–Jouguet (CJ) pressure, 2.54% higher detonation velocity and 6.60% higher CJ temperature than BCHMX. This is because nitramide group introduction increases HEDM’s nitrogen content and oxygen balance, leading to more CO_2_, N_2_ and fewer carbon clusters at the CJ state. After expansion to normal conditions from the CJ state, fewer CO gases were produced, indicating that BCHMX-ENO is more environmentally friendly than BCHMX. This study uncovers how the specific functional group influences the energetic properties of HEDMs from the atomic perspective, providing useful information for designing environmentally acceptable alternatives with improved properties.

## Graphical abstract

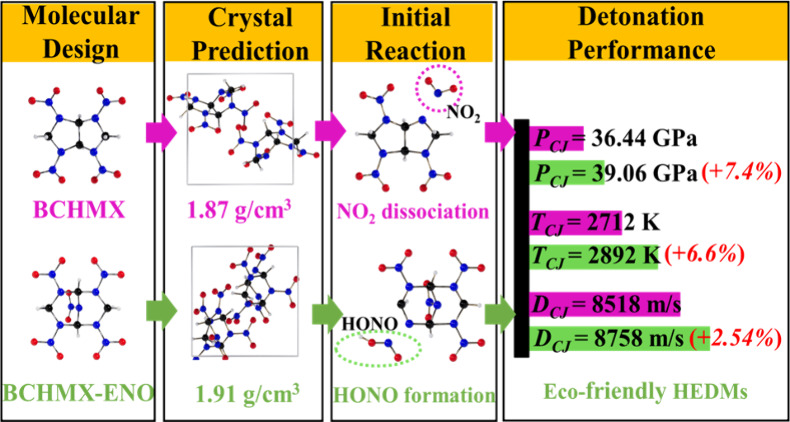

## 1 Introduction

The design and synthesis of new high energy density materials (HEDMs) with advanced performance attract great interests in many engineering applications, such as construction, mining, aerospace, and deep-sea exploration.^1,2^ Exploratory synthesizing based on current molecular structure backbones greatly enriches HEDMs’ varieties, but the inefficiency of the selection of the best structure among them comes from the complicated experimental characterization of detonation properties and products. Recently, detonation performance prediction codes with empirical fitted equation of state, such as EXPLO5,^3,4^ is becoming a popular way to evaluate newly designed HEDMs’ properties. For example, Zhang^5^ et al. predicted a detonation velocity of *V*_D_ = 9.4 km/s and a detonation pressure of *P* = 41.9 GPa for ICM-101­([2,2′-bi­(1,3,4-oxadiazole)]-5,5′-dinitramide, C_4_H_2_N_8_O_6_), which are comparable to a detonation velocity of *V*_D_ = 9.9 km s^–1^ and a detonation pressure of *P* = 40.5 GPa for TKX-50 (Dihydroxylammonium 5,5′-bistetrazole-1,1′-diolate, C_2_H_8_N_10_O_4_).^6^ Dong^7^ et al. predicted the detonation performance of C_4_N_18_H_3_^–^ anion of *V*_D_ = 8.9 km/s and *P* = 30.9 GPa, which are comparable to RDX (Hexogen, C_3_H_6_N_6_O_6_) of *V*_D_ = 8.9 km/s and *P* = 34.0 GPa.^8^ However, these codes are hardly to explain unexpected detonation properties for some new HEDMs due to the missing information on chemical and physical details in their preset detonation products’ library, especially for those complicated incomplete products during detonation processes.

Although it has been verified by many studies that the design of introducing nitrogen-rich groups into current molecular backbones is a practical method to increase the detonation properties,^9,10^ some experimental studies found that this design may also leads to low detonation performance unexpectedly. Recently, Tang et al. synthesized a FOX-7-T material (1,1-diamino-2-tetrazole-2-nitroethene) whose molecule comes by replacing one nitro group of commonly used EM of FOX-7 (1,1-diamino-2,2-dinitroethene) to a heterocyclic tetrazole ring.^11^ Compared with FOX-7, although FOX-7-T has a significantly higher nitrogen percentage, its predicted detonation performance is unexpectedly much lower. We found that the significantly incomplete combustion which leads to numerous, massive and persistently stable condensed phase carbon clusters produced at the CJ state are responsible for this low energy release efficiency phenomenon during detonating.^12^ Thus, besides energy storage density, the energy release degree, which is hardly to be characterized and easily to be neglected, is another important factor in design and synthesis of new HEDMs.

Recently, some groups found that replacing one nitro group of CL-20 to another explosophoric unit may have negative effects to the physical density.^13^ We found that CL-20s C–C bond is a good spot to introduce an ether link with comparable density from the initial structure.^14^ Thus, the position of functional group introduction within the molecule could affect the materials’ density. In addition, the molecule structure of BCHMX or bicyclo-HMX (cis-1,3,4,6-tetranitrooctahydroimidazo-[4,5-*d*]­imidazole) was designed theoretically based on the HMX (1,3,5,7-tetranitro-1,3,5,7-tetrazocane) molecule by introducing intramolecular carbon–carbon linkages, as shown in Figure 1a.^15^ Since the C–C bond serves as the shared chemical bonds of two imidazole rings with a dihedral angle of ∼75°,^16^ it provides an excellent spot to introduce extra functional groups. In this paper, we first designed a new BCHMX-ENO (2,4,6,8,9-pentanitro-2,4,6,8,9-pentaazabicyclo[3.3.1]­nonane) molecule by adding a nitramide (N-NO_2_) group into C–C bond of BCHMX, leading to an increased nitrogen percentage and a better oxygen balance, as shown in Figure 1b. Then, we predict the new molecule’s crystal structure by a method combining evolutionary algorithms (EAs) search program and density functional theory (DFT). Next, we examined the initial decomposition reaction mechanisms using QM-MD (quantum mechanics molecular dynamics) cook-off simulations on the BCHMX and BCHMX-ENO systems as a function of temperature from 300 to 3000 K. To predict the detonation parameters and products at the Chapman–Jouguet (CJ) state, we applied a method combining RxMD (reactive molecular dynamics with ReaxFF force field) and QM-MD to achieve the long convergence process from an inert material to the equilibrated detonated state. Therefore, our series simulations provide a comprehensive first-principles-based description of the full sequence of complex reactive processes including the initial decomposition reactions and the final hot dense detonated state of BCHMX and BCHMX-ENO systems.

**Figure 1:**
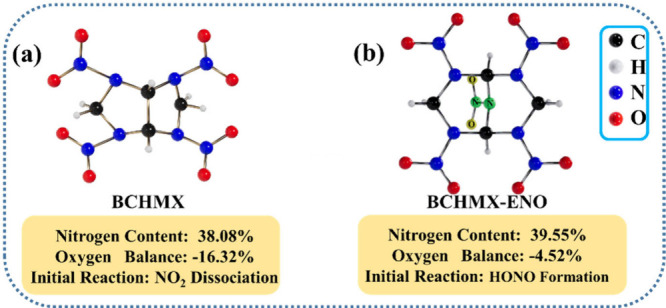
Molecular structure, nitrogen content, oxygen balance, and initial reaction of (a) BCHMX and (b) BCHMX-ENO.

## 2 Simulation Methods

### 2.1 Molecule Design and Method to Predict the Crystal Structure of BCHMX-ENO

The initial unit cell structure of BCHMX was taken from the Cambridge Structural Database^17^ and then was optimized by the PBE-D3 flavor of density functional theory (DFT). The optimized cell parameters are *a* = 8.59 Å, *b* = 6.95 Å, *c* = 8.95 Å, α = γ = 90°, and β = 102.09° (at 0 K), leading to a density of 1.87 g/cm^3^, in agreement with the X-ray experimental values^18^ of *a* = 8.59 Å, *b* = 6.95 Å, *c* = 8.97 Å, α = γ = 90°, and β = 101.78° (at 150 K) with a density of 1.86 g/cm^3^. Thus, the PBE-D3 flavor of the DFT approach describes the BCHMX crystal structure very well.

We designed the new BCHMX-ENO structure with increased nitrogen percentage and oxygen balance by adding the -N-NO_2_ group to the C–C bonds of the BCHMX molecule. Since the CHMX unit cell includes 2 molecules, we set 2 BCHMX-ENO molecules in a cell as the starting point. The search for stable phases of BCHMX-ENO crystals was carried out using the universal structure predictor: evolutionary xtallography^19^ in USPEX 9.4.4 package. In our searches, the initial population included 20 structures, followed by subsequent structures produced by heredity (40%), random structure generator (30%), softmutation (10%), and rotational mutation (20%), as illustrated in Figure 2. The minimum intermolecular distances between various types of atoms are shown in Table S1 and the minimum distance between the geometric centers of different molecules is set as 6.0 Å. After the structures were generated, they were optimized by density functional theory (DFT) and ranked by the system energy. We applied 27 generation iterations consisting of 650 structures in total to consider as many crystal structures as possible. To verify the accuracy of crystal prediction for energetic materials by USPEX program combined with DFT optimization, we took CL-20 as an example since it belongs to nitroamine compounds family just as BCHMX and BCHMX-ENO. We examined 10 generation iterations in total, as shown in Figure S1 in Supporting Information. The predicted lowest energy structure corresponds to the thermodynamically stable γ phase, and the highest density structure among energetic favorable structures corresponds to the ε phase,^20^ as shown in Tables S2 and S6 in Supporting Information. This justifies using this method to predict the crystal structure of the BCHMX-ENO nitroamine compound accurately.

**Figure 2:**
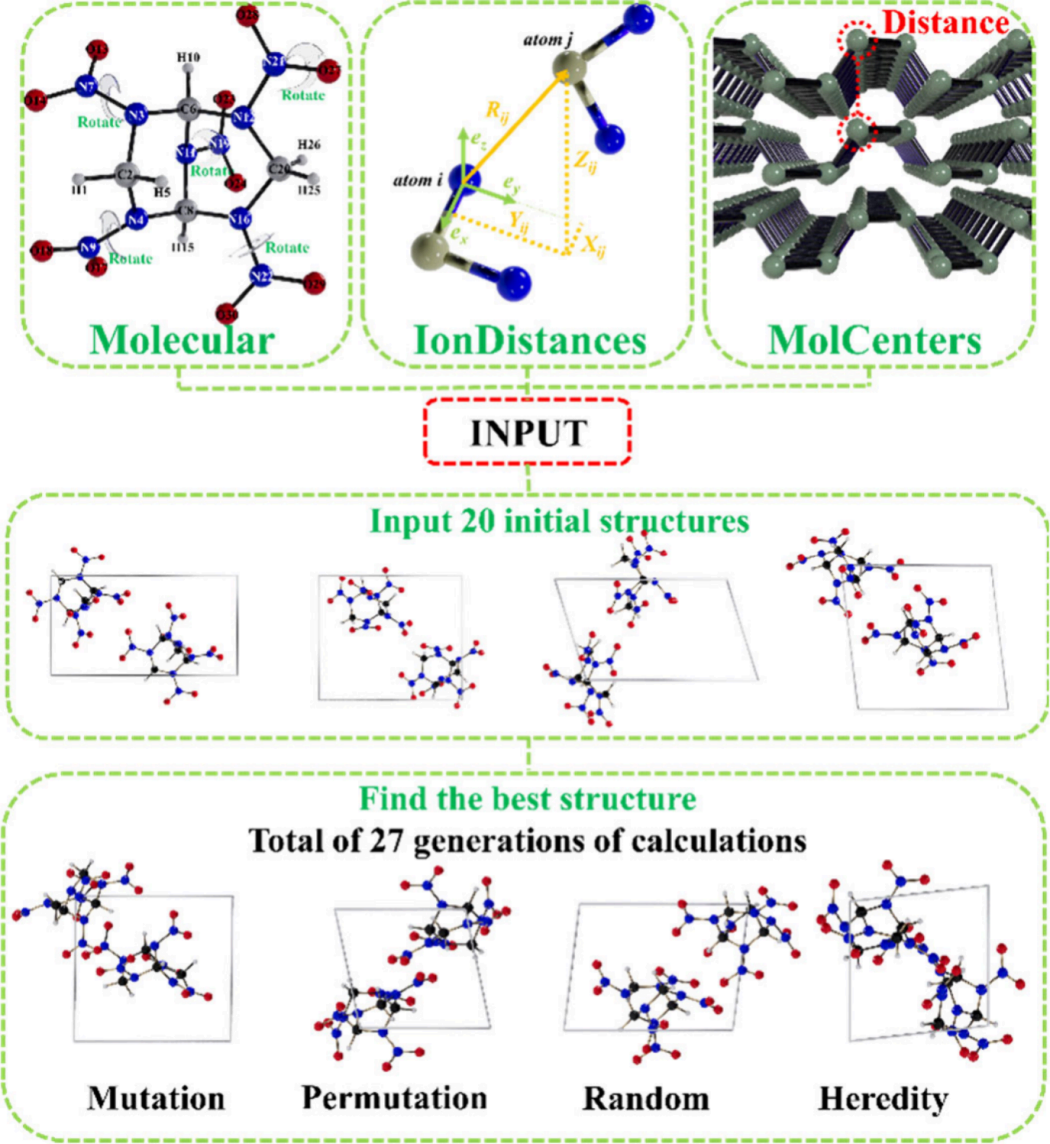
Schematic diagram of exploring stable phases of BCHMX-ENO with USPEX.

Density functional theory (DFT) simulations were conducted using the VASP (Vienna Ab initio Simulation Package) software.^21,22^ The Perdew–Burke–Ernzerhof (PBE) pseudopotential, a generalized gradient approximation (GGA) for exchange-correlation functionals,^23,24^ was employed to model electronic exchange and correlation effects. van der Waals interactions (London dispersion) were accounted for using a low-gradient correction.^25^ The projector augmented wave (PAW) method was utilized to represent the interactions between atomic nuclei and valence electrons.^26^ The geometric optimization was performed using the Conjugate Gradient (CG) algorithm.^27^ A plane-wave basis set was expanded to a cutoff energy of 500 eV, with an energy convergence criterion of 1 × 10^–6^ eV and an interatomic force convergence criterion of 1 × 10^–3^ eV/Å.

### 2.2 Methods to Predict the Initial Reaction and the Chapman–Jouguet State

We performed molecular dynamic simulations using the large-scale atomic/molecular massively parallel simulator (LAMMPS) code.^28^ We used ReaxFF force field^29^ which have been reported for many systems (such as RDX, HMX, TATB, and PBX)^30–34^ to simulate the long decomposition processes of energetic materials from the inert state to the final equilibrium state. The Nose–Hoover thermostat and barostat with a time step of 0.1 fs were used during the cook-off simulations. We used the Born–Oppenheimer QM-MD approach implemented in the VASP package to examine the initial decomposition reactions during cook-off simulations. We first heated the cells at a constant rate from 10 to 300 K in 2 ps. Then, the systems were equilibrated at 300 K for 1 ps by using the NVT ensemble (constant volume, constant temperature, and constant number of atoms) by using the Nose–Hoover thermostat. Finally, the system was heated from 300 to 3000 K at a constant heating rate of over 20 ps. A time step of 1.0 fs was employed to integrate the equations of motion for all atoms in the simulations.

We applied an empirical model proposed by Mathieu^35^ to evaluate the H_50_ in the standard drop-hammer test based on the formula as follows:

$$H_{50}/cm={\left(\frac{k_{c}}{k_{pr}}\right)}^{4}$$ 1

2 $$\log H_{50}=\frac{w_{1}a+w_{2}b+w_{3}c+w_{4}d+w_{5}DSSP+w_{6}ISSP}{molecular\;weight\;of\;the\;compound}$$

where *k*_*c*_, *w*_1_ to *w*_6_, DSSP (decreasing sensitivity structural parameters) and ISSP (increasing sensitivity structural parameters) are empirical parameters or coefficients, and *k*_pr_ represents the kinetic parameter associated with the expansion step in the decomposition process. a,b,c,d denote the stoichiometric numbers of carbon, hydrogen, nitrogen, and oxygen atoms in the compound C_a_H_b_N_c_O_d_.

We applied a method combining RxMD and QM-MD to locate the Chapman–Jouguet (CJ) point and calculate the CJ properties.^36^ The detonation performance of HEDMs can be elucidated by classical Zeldovich–von Neumann–Döring (ZND) and Chapman–Jouguet (CJ) theory.^37^ The ZND detonation model^38^ defines that a detonation wave comprises a shock front with a just followed chemical reaction zone. Although the speed of the shock wave might be varied when propagating in the reacted material, the CJ theory^39^ defines that only one velocity satisfies the condition of the self-sustained detonation due to the limits of stored chemical energy in the local material. The chemical equilibrium state at the end of the reaction zone for the sustained detonation is denoted as the CJ state.^40^ By applying the principles of mass and momentum conservation before and after the detonation wave, the Hugoniot equation of the material can be expressed as

3 $$Hg=e-e_{0}-\frac{1}{2}\left(p+p_{0}\right)\left(v_{0}-v\right)$$

where *p* represents pressure, *e* is the specific internal energy, and *v* signifies specific volume. Here, the term “specific” refers to the quantity per unit mass, and the subscript “0” pertains to the initial inert state. The CJ point is located in the tangency between the Hugoniot curve of the detonation products and the Rayleigh line describing the shock wave propagation in the material.

In eq 3, the initial state’s parameters were obtained from QM-MD of the equilibrated inert system at 300 K. A completely detonated state was achieved first through a ∼150 ps RxMD simulations with a specific density and temperature, which describe the whole chemical dissociation processes from the inert initial crystal to final products. Then, a 20 ps QM-MD of the equilibrated detonated system was performed based on the last step of RxMD trajectories, and the first-principles description of detonation parameters were obtained by averaging the thermodynamic parameters of the last 10 ps simulation. Twenty RxMD & QM-MD calculations in total from five different sets of temperatures with four different densities for each temperature were performed to achieve a series of Hugoniot values (Hgs). These Hgs were fitted using the spline function as five groups of isotherms on the Hg-density plane. Since only the Hg = 0 states satisfy the rule of energy conservation before and after the detonation shock wave, the locations of the intersections between Hugoniot values and the Hg = 0 axis give the compression volume ratios for the detonated Hugoniot curve in the pressure–volume (*P*–*V*) plane. The pressure and volume of these five Hg = 0 points were fitted into a quadratic polynomial in the *P*–*V* plane. The CJ point is the tangent point between the Hugoniot curve and the Rayleigh line which describes the shock wave propagation. Finally, the detonation velocity was calculated based on the CJ pressure, and the CJ temperature was derived from the *T*–*V*/*V*_0_ quadratic polynomial curve.

We analyzed the complex reactions with the bond order cutoff values listed in Table S3 in the Supporting Information. These values were optimized to have a good description of fragments during chemical reactions based on our previous simulations of energetic material systems.^12,14,36^

As a comparison, we also performed EXPLO5 V6.04.02 thermochemical code^3^ to predict the CJ state. To predict the detonated products state, it employs the Becker–Kistiakowsky–Wilson (BKW) equation, which is expressed as

4 $$\frac{pv}{nRT}=1+xe^{\beta x}$$

5 $$x=\frac{k}{v{\left(T+\theta \right)}^{\alpha }}$$

6 $$k=\gamma \sum x_{i}k_{i}$$

where *x*_*i*_ is the mole fraction of the *i*-th gaseous product, *k*_*i*_ is the molar covolume of the *i*-th gaseous product, *T* is the temperature, *p* is the pressure, *v* is the molar volume of the gas, and *R* is the gas constant. and *n*, α, β, γ and θ are empirical constants.

EXPLO5 program includes a database of preset reactants and products. To predict the detonation product distribution, a freeze point within the temperature range of 1500 to 1800 K was defined by freezing chemical reactions and detonation products. Then the CJ parameters were predicted by locating the CJ point based on the expansion isentrope and the shock adiabat.

The oxygen balance (OB) is an index of the accessibility of fuel atoms to oxygen atoms in an energetic material system. Since it is a critical parameter to evaluate the deficiency or excess of oxygen required to convert fuel atoms (hydrogen and carbon) into final products (water and carbon dioxide), it determines the energy release efficiency of an energetic material. The formula for calculating OB for C_a_H_b_N_c_O_d_ can be expressed as

7 $$OB\;\left(\%\right)=\frac{1600\times \left[d-\left(2a+\frac{b}{2}\right)\right]}{molecular\;weight\;of\;the\;compound}$$

## 3 Results and Discussion

### 3.1 Search for Stable Phase of BCHMX-ENO Using the USPEX Method

USPEX predicted 20 initial cell structures in the first generation. These structures were optimized by density functional theory, and their energies were ranked from the lowest to the highest. Then, 40% of energetic favorable crystal systems were inherited and adjusted, and 60% of new structures were produced by random structure generator, softmutation, and rotational mutation algorithms, leading to 25 structures in the second generation. However, not all of the predicted structures were physically realistic. Those unreasonable structures would be deleted once geometry optimization fails. As a result, 20 to 25 structures were retained finally for each generation, leading to a total number of 650 for 27 generations. The population of crystal structures in each iteration is listed in Table S4 in Supporting Information. The relative molecular energy differences for each structure are shown in Figure 3. The predicted lowest energy structures from all generations are shown in Figure S2.

**Figure 3:**
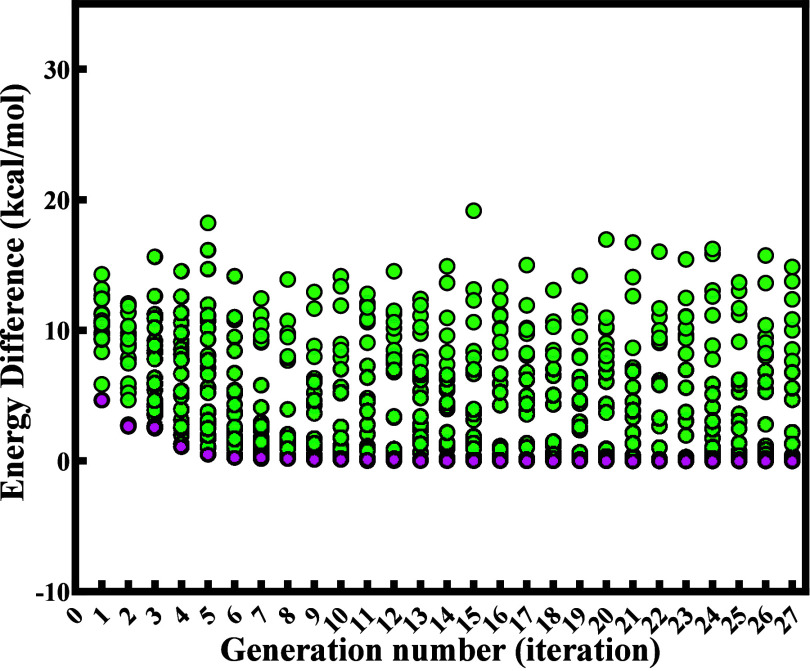
Relative energies of BCHMX-ENO molecular structures predicted by USPEX of all generations. The red dots represent the structure with the lowest energy in each iteration. The lowest energy structure was set as the reference.

We considered 20 energetic favorable structures (listed in Table S5) as the most possible candidates because their energy differences fall in a very small range of 0.2 kcal/mol/molecule. We are interested in the BCHMX-ENO crystal structure (as shown in Figure 4) with a cell parameter of *a* = 8.66 Å, *b* = *c* = 8.44 Å, α = γ = 90°, and β = 87.41° because of its high density of 1.91 g/cm^3^.

**Figure 4:**
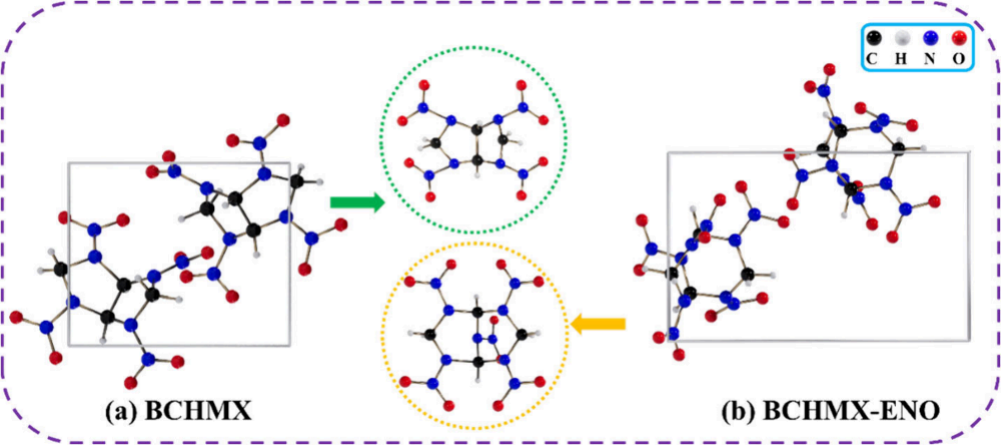
Structures of the unit cells and single molecules of (a) BCHMX and (b) BCHMX-ENO.

### 3.2 Sensitivity Evaluation and Reaction Mechanisms of BCHMX and BCHMX-ENO

The evaluation of sensitivity and determination of decomposition mechanism are essential to energetic systems because the initial decomposition indicates a risk of enormous energy release,^36,41^ so we consider static molecular stability, dynamic thermal stability and impact sensitivity to comprehensively evaluate the sensitivity of BCHMX and BCHMX-ENO.

Since the HOMO–LUMO gap reflects the external energy required for electron excitation of a molecule, we calculated the gap energy to examine their static molecular stability, as shown in Figure 5. The calculated HOMO–LUMO gaps for BCHMX and BCHMX-ENO are 0.221 and 0.211 eV, respectively. Thus, the molecular stability of BCHMX-ENO is slightly higher than that of BCHMX.

**Figure 5:**
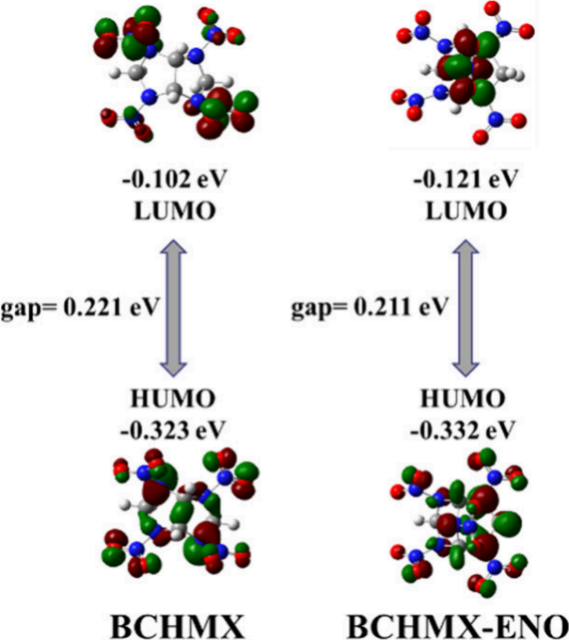
HOMO–LUMO diagram of BCHMX and BCHMX-ENO molecules.

To evaluate the dynamic thermal stability of BCHMX and BCHMX-ENO, we started with the experimental unit cell of BCHMX and the predicted cell of BCHMX-ENO. In order to examine the thermal stability and to understand the initial decomposition reaction mechanism of two systems, we analyzed the molecular fragments during the cook-off simulations with a linear increased temperature from 300 to 3000 K, as shown in Figure 6. To verify the accuracy of QM-MD simulations, we examined the chemical reactions of β-HMX with the same cook-off procedure and compared it with BCHMX. We found the first reaction of the β-HMX system occurs at 2230 K (as shown in Figure S4 in Supporting Information), which is higher than 2217 K of the BCHMX system. Thus, our results agree well with the Klasovitý et al.’s experimental DTA observation.^15^

**Figure 6:**
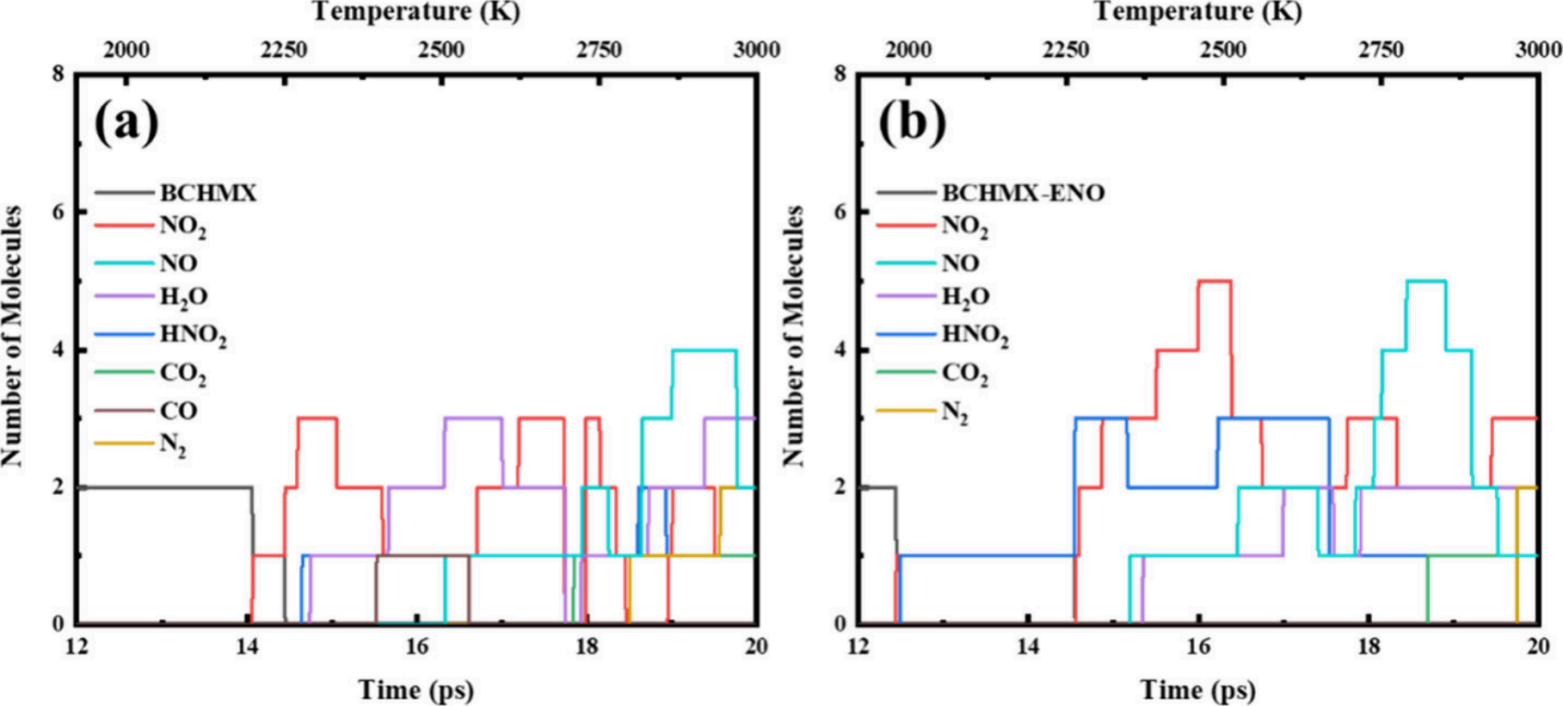
Species analysis for the decomposition of (a) BCHMX and (b) BCHMX-ENO heated from 300 to 3000 K over 20 ps.

For BCHMX, no reactions were observed until ∼2200 K, as shown in Figure 6a. The first reaction was a unimolecular decomposition at 2217 K with one molecule releasing a NO_2_. Then we found one molecule dramatically deformed with two C–N bonds stretched at the same time, further leading to the destruction of bicyclic ring into fragments including a single five-membered ring, a NO_2_ and a N_2_C_4_H at 2250 K. The second molecule also decomposed in the sequence of NO_2_ dissociation followed with the ring rupture, as shown in Figure 7. Thus, NO_2_ cleavage is the first reaction step in the initial thermal decomposition reactions, agreeing with our previous studies.^17^ HONO formation was rarely seen before 2277 K but commonly at higher temperatures. Final product molecules, such as water, carbon dioxide, and nitrogen gas, appeared initially at 2291, 2710, and 2799 K, respectively.

**Figure 7:**
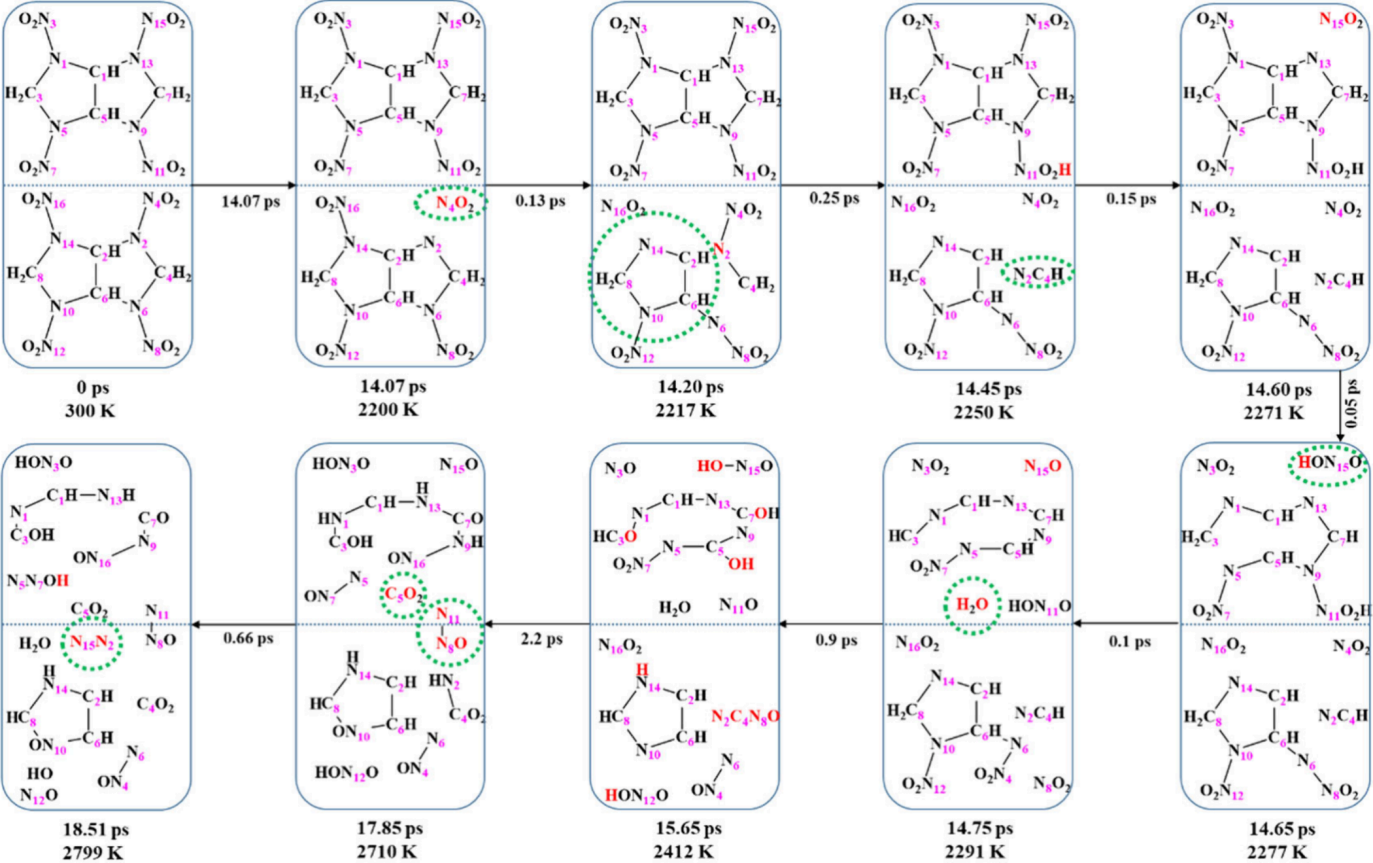
Decomposition process of BCHMX during 300–3000 K heating.

For BCHMX-ENO, we found that the initial reaction of NO_2_ dissociation occurred at a temperature of 1980 K during the heating process, as shown in Figure 6b. This NO_2_ turns into HONO immediately by attacking one of the two protons from the neighbor carbon atom, as shown in Figure 8. Similarly, two more HONOs from two independent molecules were observed at 2264 K. Thus, unimolecular HONO formation is the key reaction mechanism in the initial thermal decomposition of BCHMX-ENO, providing the starting point for subsequent decomposition reactions. Then a NO_2_ were produced due to the destruction of the bicyclic ring with two C–N bonds break at 2271 K. At this moment, NO_2_ has a hard time to rob another proton from surrounding carbon atoms to form HONO because each carbon only owns one hydrogen atom. As the temperature increased, more complex reactions were started. The water, carbon dioxide, and nitrogen gas molecules appear first at 2372, 2824, and 2966 K, respectively. Thus, the BCHMX-ENO system shows a slightly higher thermal sensitivity than BCHMX. However, BCHMX-ENO’s initial reaction temperature is comparable to many other thermal stable systems that we studied previously, such as MTO (1800 K)^42^ and TKX-50 (1700 K)^43^ and 4,4′-bis­(dinitromethyl)-3,3′-azofurazanate MOF (1970 K).^44^ Thus, the strategy of introducing a -N-NO_2_ group into the CC bonds is a promising way to achieve new nitramine EMs with a reasonable stability.

**Figure 8:**
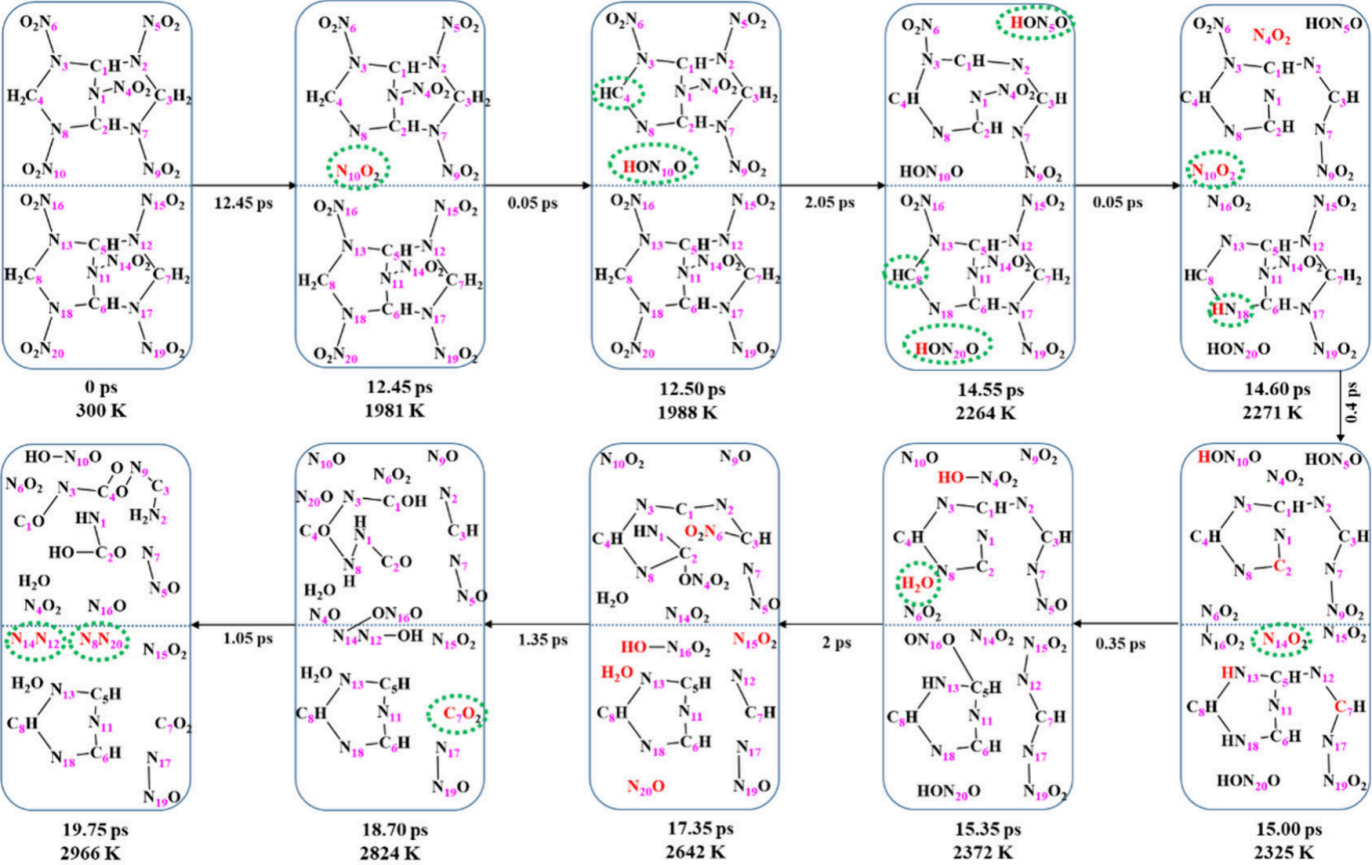
Decomposition process of BCHMX-ENO molecules during 300–3000 K heating.

To understand the different initial intramolecular reaction steps between BCHMX and BCHMX-ENO, we measure the N–N bond length and the distance between the O of the nitro group and the H of the methylene group for each molecule at 0 K, as shown in Figure 9. For BCHMX, the average O–H distance is 2.469 Å and the average bond length of N–N is 1.382 Å; however, for BCHMX-ENO, the O–H distance decreases to 2.296 Å, but the average N–N bond length increases to 1.402 Å. This is because the inserted N-NO_2_ group changes the spatial distribution of the molecule configuration. During heating, we speculate that a longer N–N bond facilitates NO_2_ dissociation, and a shorter O–H distance facilitates proton transfer from carbon to oxygen, leading to an easier initial NO_2_ decomposition and more HONO formation in BCHMX-ENO system than those in BCHMX system at initial stage. Our finding that longer average N–N bond length leads to lower stability agrees with Klasovitý et al.’s DTA experiments of BCHMX and β-HMX.^15^

**Figure 9:**
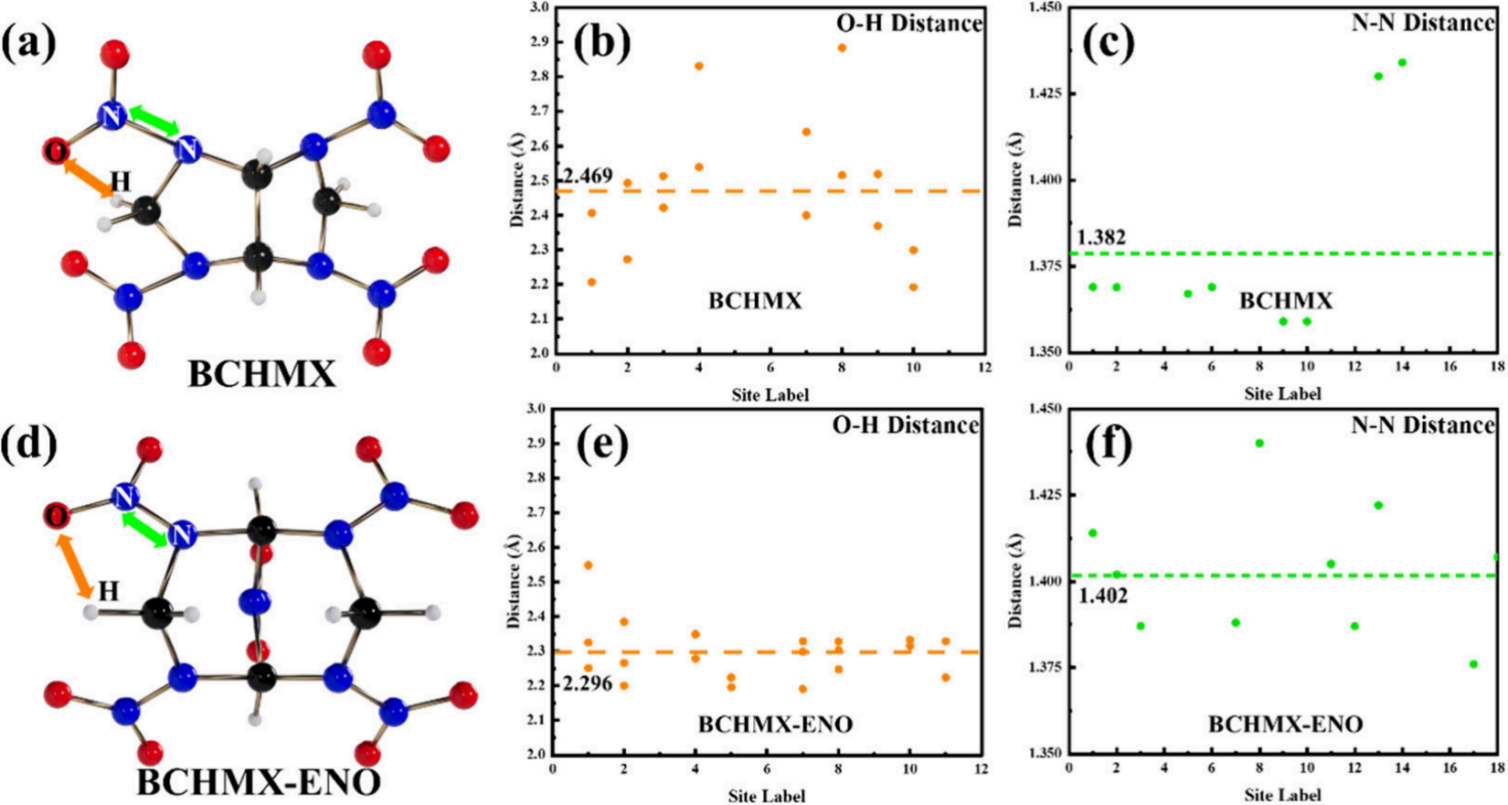
Molecular structures of (a) BCHMX and (d) BCHMX-ENO. (b, e) H–O bond distance and (c,f) N–N bond length for BCHMX and BCHMX-ENO molecule, respectively.

To evaluate the impact sensitivity of BCHMX and BCHMX-ENO, we applied an empirical model proposed by Mathieu^35^ to evaluate the H_50_ in the standard drop-hammer test, based on eq 1. We first calculated HMX’s H_50_ value as a benchmark to verify the equation’s accuracy. The calculated value of 12.9 cm matches well with the experimental value of 12.0 ± 0.5 cm.^45^ Thus, we believe this model could provide impact sensitivity of BCHMX and BCHMX-ENO at the same accuracy level. The calculated H_50_ values of BCHMX and BCHMX-ENO are 13.7 and 12.3 cm, respectively. Thus, the impact sensitivity of BCHMX-ENO is comparable to HMX and slightly higher than BCHMX.

### 3.3 Detonation Performance of BCHMX and BCHMX-ENO

The CJ state describes a specific high-pressure and high-temperature state of a sustained detonation propagating in a reacted material, and its mathematic description locates somewhere in the equation state of chemical equilibrated detonation products in the pressure–volume (*P–V*) plane.^46^ To achieve an accurate first-principles description of a completely reacted state, we first performed long cook-off RxMD simulations at high temperatures using ReaxFF, following with extra QM-MD simulations based on the atomic trajectories and velocities of the last RxMD step. ∼150 ps RxMDs were carried out to achieve the equilibrated detonated state because the total energies of both BCHMX and BCHMX-ENO systems keep constant after 75 ps. ∼20 ps QM-MDs were then performed to further verify the unchanged system energy and to accurately describe the state of the decomposition products. The properties of the detonated systems were calcualted based on the average values of last 10 ps QM-MD simulations. The total energy evolution of BCHMX system at 3000 K with a compression ratio of 0.75, and of BCHMX-ENO system at 2600 K with a compression ratio of 0.65 were shown in Figure S3.

The pressures of detonated states in the *P*–*V* plane are dependent on two factors of temperature and density.^47^ Thus, we tested and adjusted a series of combinations of temperatures and densities to satisfy Hugoniot values close to zero. For a specific temperature, an isotherm was obtained by spline fitting a group of four Hugoniot values based on various densities. Five isotherms were then derived based on five temperatures from 20 cook-off simulations in total. Hg = 0 represents the energy conservation before and after the detonation wave, so five sets of thermodynamic parameters (*V/V*_0_, *T*) from the intersections of the isotherms with the Hg = 0 axis locate the volume compression ratios at corresponding temperatures of the fully reacted Hugoniot curve, as shown in Figure 10.

**Figure 10:**
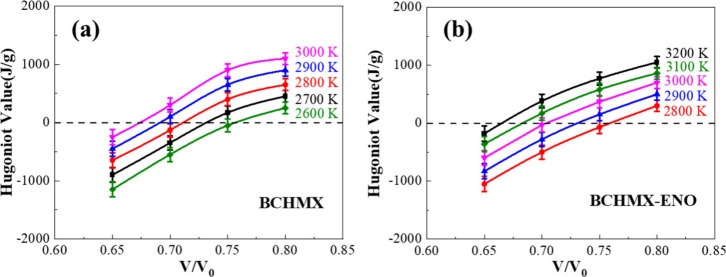
Spline-fitted curves of Hugoniot values and the volume-compression ratios at different temperatures for (a) BCHMX and (b) BCHMX-ENO. The positions of fully reacted Hugoniot states are determined by the intersections of the Hg = 0 line (dashed line) with five isotherms.

The detonation Hugoniot curves of BCHMX and BCHMX-ENO were determined by quadratic polynomial fitting the pressures of detonated states based on the *V/V*_0_ parameters satisfying Hg = 0 on the *P–V* plane. The location of CJ point is determined by a tangent Rayleigh line^48^ which describes the propagation state of sustained detonation wave from (1,0) point to the detonation Hugoniot curves,^49^ as shown in Figure 11a,c. Once the CJ location was found, the CJ pressure could be derived from the determined detonation velocity. Also, the relationship between temperature and volume compression ratio is established to determine the temperature at the CJ state (Figure 11b,d).

**Figure 11:**
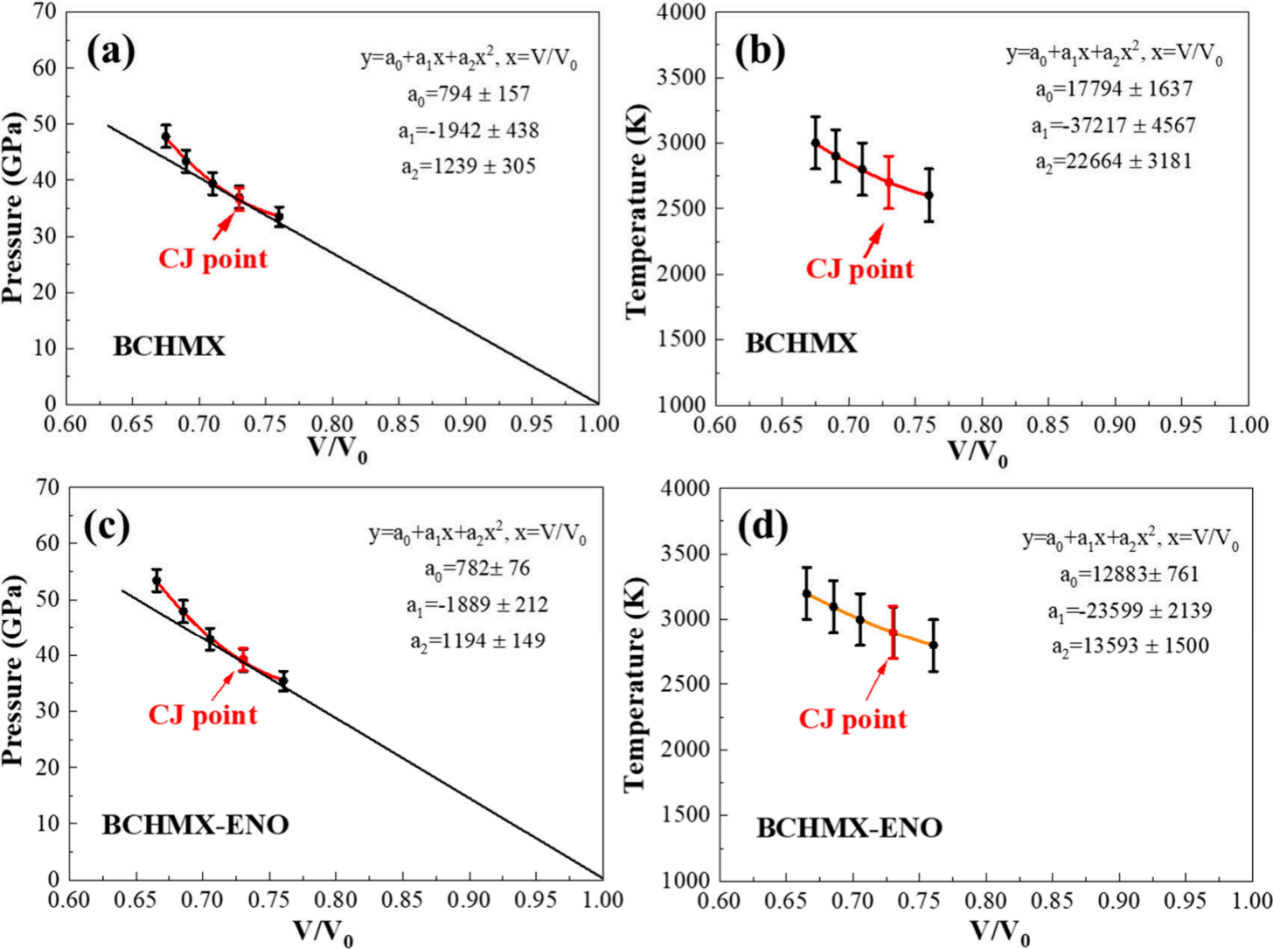
Hugoniot curve and CJ point for (a) BCHMX and (c) BCHMX-ENO. (b, d) CJ temperature of BCHMX and BCHMX-ENO. The CJ points (indicated by red dots) are determined as the points of tangency between the Rayleigh line and the fully reacted Hugoniot curve.

For BCHMX, the predicted detonation properties by RxMD&QM-MD and EXPLO5, and two sets of experimental data are listed in Table 1. A Cheetah report conducted by Elbeih^50^ et al. was performed as a comparison. The predicted detonation velocity from our RxMD&QM-MD is 8.537 ± 0.232 km/s at an initial density of 1.86 g/cm^3^, which compares well with the experimental data of 8.65 km/s and 8.70 km/s at a density of 1.79 g/cm^3^. Thus, our RxMD&QM-MD results are validated by the experimental data. The detonation velocities predicted by Cheetah and EXPLO5 are 8.832 km/s at a density of 1.79 g/cm^3^ and 9.102 km/s at a density of 1.87 g/cm^3^, respectively. The CJ pressure predicted by RxMD&QM-MD, Cheetah and EXPLO5 are 36.60 ± 2.02 GPa, 33.24 and 37.48 GPa, respectively. Thus, the energy delivery capabilities for external expansion of BCHMX predicted by three procedures reach a reasonable agreement. The divergence comes from the predicted CJ temperature with a temperature of 2712 ± 202 K from RxMD&QM-MD, which is 32.95% lower than that of EXPLO5 codes. EXPLO5 develops a way to halt all chemical reactions and freeze the products composition at 1800 K to have a comparable heat of detonation with Ornellas’ calorimetrically.^51–53^ Probably this difference arises because the product distributions assumed in EXPLO5 are different from those found in our first-principles reactive dynamics simulation.

**Table 1:** Detonation Properties Predicted by RxMD&QM-MD and EXPLO5 for BCHMX, Comparing with CHEETAH Prediction and Experimental Results; Detonation Properties Predicted by RxMD&QM-MD for BCHMX-ENO

	**BCHMX**					**BCHMX-ENO**
	**RxMD** **&QM-MD**	**EXPLO5**	**CHEETAH** ^50^	**EXP** ^54^	**EXP** ^18^	**RxMD** **&QM-MD**
**Density (g/cm** ^**3**^ **)**	1.86	1.87	1.79	1.79	1.79	1.90
***P***_**CJ**_ **(GPa)**	36.60 ± 2.02	37.84	33.24			39.31 ± 2.04
***D***_**CJ**_ **(km/s)**	8.537 ± 0.232	9.102	8.832	8.65	8.70	8.754 ± 0.224
***T***_**CJ**_ **(K)**	2712 ± 202	4045				2892 ± 218

For BCHMX-ENO, the predicted detonation velocity by RxMD&QM-MD is 8.754 ± 0.224 km/s which is 2.54% higher than BCHMX system, while the predicted CJ pressure is 39.31 ± 2.04 GPa which is 7.40% higher. We expect those are because more nitrogen gases due to the increased nitrogen content and smaller portions of masses in aggregates due to the improved oxygen balance at CJ state in BCHMX-ENO system than in BCHMX system.^15^ The predicted CJ temperature is 2892 ± 218 K which is 6.6% higher than for BCHMX, indicating more fully reacted gas phase products are generated at the CJ state. Thus, the detonation properties are predicted to be enhanced by introducing a -N-NO_2_ group into the BCHMX system.

To gain a detailed atomistic understanding of why BCHMX-ENO exhibits enhanced detonation properties compared to BCHMX, we first analyzed the CJ products of QM-MD simulations as shown in Table 2. Compared with BCHMX system, BCHMX-ENO system has similar species of the main CJ gaseous products, but the amount for each is significantly increased. For BCHMX, the quantities of N_2_, CO_2_, H_2_O, and CO at the CJ states are 3.08 ± 0.02 mol/mol, 0.39 ± 0.03 mol/mol, 0.08 ± 0.02 mol/mol, and 0.45 ± 0.04 mol/mol, respectively; while for BCHMX-ENO, the quantities are 4.50 ± 0.00 mol/mol, 1.60 ± 0.03 mol/mol, 0.50 ± 0.05 mol/mol, and 0.50 ± 0.00 mol/mol, respectively. These increased yields of fully reacted gases lead to increased energy release at the CJ state, explaining why BCHMX-ENO shows a higher CJ temperature than BCHMX. On the other hand, similar average composition of carbon clusters was observed in both two systems (C_1.58_H_1.24_N_0.58_O_1.59_ for BCHMX compared with C_1.58_H_1.47_N_0.83_ O_2.22_ for BCHMX-ENO), but less masses (0.37 g/g in BCHMX compared with 0.23 g/g in BCHMX-ENO) were found in BCHMX-ENO system. Since less percentage of masses were trapped in carbon clusters in the BCHMX-ENO system, the CJ pressure and detonation velocity are increased.

**Table 2:** CJ Products and Detonation Products Predicted by RxMD&QM-MD and EXPLO5 for BCHMX and BCHMX-ENO

		**BCHMX**			**BCHMX-ENO**	
		***RxMD** **&QM-MD***		***EXPLO5***	***RxMD** **&QM-MD***	
		**CJ products**	**Detonation products**	**CJ products**	**CJ products**	**Detonation products**
**main products**(mol/mol)	**N**_**2**_	3.08 ± 0.02	4.00	3.98	4.50 ± 0.00	5.00
	**CO**_**2**_	0.39 ± 0.03	2.00	1.32	1.60 ± 0.03	3.50
	**H** ^**+**^	2.37 ± 0.13			2.03 ± 0.18	
	**H**_**2**_		1.00	0.01		0.50
	**CO**	0.45 ± 0.04	2.00	0.72	0.50 ± 0.00	0.50
	**H**_**2**_ **O**	0.08 ± 0.02	2.00	1.23	0.50 ± 0.05	2.50
	**HO** ^**–**^	0.58 ± 0.05			0.97 ± 0.09	
	**CH**_**2**_ **O**_**2**_			1.70		
	**NH**_**3**_			0.03		
	**HCN**			0.01		
**Other molecules**(g/g)						
**Noncarbon gases**	0.19	None		0.10	None	
**Composition**	H_0.14_N_0.22_O_0.95_			H_0.12_O_1.12_		
**Carbon clusters**	0.37	None	0.83	0.23	None	
**Composition**	C_1.58_H_1.24_N_0.58_O_1.59_		Diamond	C_1.58_H_1.47_N_0.83_O_2.22_		
**Carbon cluster material recovery (%)**						
**C**	78.95			47.50		
**H**	41.23			29.44		
**N**	14.47			10.00		
**O**	39.80			26.67		

We analyzed the CJ products of the EXPLO5 program, as a comparison. Only two major carbon compounds with simple compositions of CH_2_O_2_ and diamond were found at the CJ state. This leads to more final gaseous products, such as 3.98 mol/mol N_2_, 1.23 mol/mol H_2_O, 1.32 mol/mol CO_2_ and 0.72 mol/mol CO. Thus, the simplified carbon clusters and more final products lead to a higher predicted CJ temperature of EXPLO5 codes than that of RxMD&QM-MD.

To examine the environmental impact of these two systems, we predicted the final detonation products by implementing a “linear volume expansion” approach with variable-volume RxMD simulations to mimic the expansion process beyond the CJ point until ambient pressure state as in the calorimetric experiment. The initial system volume *V*_0_ was gradually expanded at a linear rate, reaching 8*V*_0_ over 20 ps and reducing the internal pressure to approximately 1 atm. Then, we obtained a QM-MD description through a 1 ps simulation starting with atom positions and velocities in the last step of the RxMD simulations. This expansion process can examine the stability of carbon aggregates and achieve the final detonation products, allowing for a direct comparison to the detonation products in the calorimetric experiment.

For both systems, we found that all of the agglomerates decompose into gases, indicating that fuel atoms in carbon clusters are accessible to oxygen atoms. In the BCHMX system, besides a large portion of masses that are in final reacted gases, such as 4.00 mol/mol N_2_, 2.00 mol/mol CO_2_ and 2.00 mol/mol H_2_O, a considerable amount of incomplete burning products of CO (2.00 mol/mol) and H_2_ (1.00 mol/mol) are formed. This is attributed to a shortage of oxygen atoms to partial carbon and hydrogen atoms to form fully oxidized gases. With the nitramino group introduced, 1.50 mol/mol less CO and 0.50 mol/mol less H_2_ were produced in the BCHMX-ENO system, leading to a higher detonation performance than BCHMX. Thus, the strategy of introducing -N-NO_2_ group into the CC bonds is a promising way to achieve new more environmental acceptable nitramine EMs with an increased performance.

## 4 Conclusions

In this work, we first employed evolutionary algorithms USPEX to predict the crystal structure of our newly designed BCHMX-ENO system. Then, we employed QM-MD simulations to examine the initial thermal decomposition reactions and used a combination of RxMD and QM-MD simulations to predict the detonation performance of BCHMX and BCHMX-ENO. The key points from our simulations are as follows:

- The spatial distribution of the molecule configuration plays a significant role in the initial decomposition reaction. For BCHMX, NO_2_ dissociation is the initial decomposition reaction, following with a ring-breaking reaction from two C–N bonds; for BCHMX-ENO, HONO formation dominates the initial steps. The slight lower thermal stability of BCHMX-ENO comes from the average longer N–N bond and shorter O–H distance of the molecule.
- Compared to BCHMX, BCHMX-ENO exhibits an enhanced energy release and external expansion capability including 7.40% higher CJ pressure, 2.54% higher detonation velocity and 6.60% higher CJ temperature. This is because compared with BCHMX system, an increased fraction of carbon atoms in the BCHMX-ENO system are accessible to oxygen atoms to form more oxidized carbon oxides with fewer carbon clusters at the CJ state, freeing more nitrogen atoms to form nitrogen gases. Thus, the oxygen balance and nitrogen percentage influence the detonation performance by affecting the composition of carbon aggregates and the amount of N_2_ gases at the CJ state, respectively.
- For both systems, all agglomerates decompose into gases after adiabatic expansion. However, BCHMX-ENO is more environmentally friendly than BCHMX because few toxic CO gases were produced after detonating.

Our results suggest that introducing a nitramide group into a C–C bond is a promising way to design the next generation of “green” HEDMs with reasonable thermal stabi lity and excellent detonation performance.

## Supplementary Material



## Notes

Ion distance matrices for different types of atoms (Table S1); CL-20/BCHMX-ENO structures predicted by USPEX with enthalpy/density/lattice parameters­(Table S2 and S5); bond order cutoffs for chemical reactions (Table S3); optimization progress of BCHMX-ENO structures across iterations (Table S4); comparison of the CL-20 crystal structures predicted by USPEX and experimental data (Table S6); relative energy distribution of CL-20 structures from USPEX generations (Figure S1); lowest-energy BCHMX-ENO structures from 27 USPEX generations (Figure S2); time evolution of total energy per unit mass during cook-off simulations for BCHMX and BCHMX-ENO systems (Figure S3); and species analysis for β-HMX decomposition under heating (Figure S4) (PDF)
