# Supplementary material for: Introducing Nitramide Group into High Energy Density Material Molecule Leads to Enhanced Performance
Source: JACS Au. 2025 Jul 2;5(7):3228–39. doi: 10.1021/jacsau.5c00411 (PMC12308372; doi:10.1021/jacsau.5c00411)
Supplement: Supplementary file 1 [file au5c00411_si_001.pdf]

Supplementary Material

Introducing Nitramide Group into High Energy Density Material Molecule Leads to Enhanced Performance

Yi Wang[1], Shichao Liu[1], Wei Le[1], Sergey V. Zybin[2], Wanjun Zhao[1], Fenglei Huang[1], William A. Goddard III[2], Dezhou Guo[1]**

[1]State Key Laboratory of Explosion Science and Safety Protection, Beijing Institute of Technology, Beijing 10081, China

[2]Materials and Process Simulation Center, California Institute of Technology, Pasadena, California 91125, United States

[*] Corresponding author-Email: wag@caltech.edu, dezhoug@bit.edu.cn.

[*]Corresponding author.

E-mail addresses: wag@caltech.edu, dezhoug@bit.edu.cn.

Table S1. The ion distance matrix for different types of atoms.

	**C**	**H**	**O**	**N**
**C**	1.70 Å	1.50 Å	1.74 Å	1.72 Å
**H**	1.50 Å	1.28 Å	1.53 Å	1.51 Å
**O**	1.74 Å	1.53 Å	1.78 Å	1.76 Å
**N**	1.72 Å	1.51 Å	1.76 Å	1.74 Å

Table S2. The 20 lowest energy structures of CL-20 predicted by USPEX.

Rank	Enthalpy (kcal/mol/molecule)	Volume (Å^3)	Density (g/cm^3)	Space group	Cell parameters					
					a	b	c	α	β	γ
1	0.000	1465.982	1.981	$Pca2_1$	13.282	8.233	14.823	90	109.17	90
2	0.046	1474.567	1.997	$P2_12_12_1$	11.471	11.063	12.738	90	90	90
3	0.078	1475.239	1.973	$Pca2_1$	8.651	12.103	13.878	90	105.72	90
4	0.085	1475.069	1.973	$P2_12_12_1$	13.321	8.172	14.873	90	90	90
5	0.129	1476.854	1.971	$P2_12_12_1$	13.352	8.204	14.833	90	90	90
6	0.176	1476.803	1.971	$P2_12_12_1$	7.808	14.809	13.027	90	90	90
7	0.179	1476.791	1.971	$P2_12_12_1$	11.527	12.365	11.111	90	90	90
8	0.183	1476.516	1.971	$P2_1/n$	8.466	10.087	17.329	90	90	90
9	0.207	1477.324	1.97	$P2_12_12_1$	8.601	10.101	17.021	90	90	90
10	0.239	1477.503	1.97	$P2_12_12_1$	8.590	10.097	17.032	90	90	90
11	0.343	1477.551	1.97	$Pca2_1$	8.561	10.154	17.150	90	107.58	90
12	0.453	1468.991	1.969	$P2_1/n$	8.534	10.07	17.191	90	90	90
13	0.589	1478.252	1.969	$Pca2_1$	8.593	10.078	17.038	90	106.73	90
14	0.612	1477.802	1.969	$P2_12_12_1$	8.862	10.316	17.657	90	90	90
15	0.716	1479.273	1.968	$Pca2_1$	9.672	13.001	11.644	90	105.63	90
16	0.821	1479.394	1.967	$P2_1/n$	8.408	12.177	14.368	90	90	90
17	1.354	1485.703	1.959	$P2_1/n$	8.348	12.157	14.579	90	90	90
18	1.359	1446.222	2.032	$Pca2_1$	8.522	12.553	13.381	90	106.82	90
19	1.387	1448.221	2.022	$Pca2_1$	7.822	12.589	14.223	90	104.58	90
20	1.806	1448.752	2.044	$Pca2_1$	8.850	12.566	13.386	90	105.45	90

Table S3. Bond Order Cutoff Values for Various Atom Pairs

	**C**	**H**	**O**	**N**
**C**	0.55	0.40	0.80	0.50
**H**		0.55	0.40	0.55
**O**			0.65	0.55
**N**				0.45

Table S4. The number of successfully optimized structures in each iteration of BCHMX-ENO.

Iterations	1	2	3	4	5	6	7	8	9	10	11	12	13	14
Structure Population	20	24	25	25	25	25	25	25	25	22	25	24	25	25
Iterations	15	16	17	18	19	20	21	22	23	24	25	26	27	
Structure Population	25	25	25	22	25	25	22	25	22	22	25	25	22	

Table S5. The 20 lowest energy structures of BCHMX-ENO predicted by USPEX.

Rank	Enthalpy (kcal/mol/molecule)	Volume (Å^3)	Density (g/cm^3)	Space group	Cell parameters					
					a	b	c	α	β	γ
1	0	621.019	1.894	P1	8.07	11.209	6.965	90	82.95	83.17
2	0.023	620.688	1.895	P1	8.043	11.247	6.963	90	82.94	83.24
3	0.034	621.201	1.893	P1	8.061	11.269	6.948	90	83.1	82.53
4	0.046	619.469	1.899	P1	8.051	11.229	6.956	90	82.92	83.13
5	0.057	619.655	1.898	P1	8.071	11.211	6.951	90	83.05	83.04
6	0.069	620.566	1.895	P1	8.043	11.285	6.947	90	83	82.59
7	0.069	618.813	1.901	P1	8.059	11.215	6.954	90	82.92	82.94
8	0.08	618.96	1.9	P1	8.051	11.253	6.942	90	82.98	82.59
9	0.08	619.057	1.9	P1	8.043	11.251	6.951	90	82.96	82.65
10	0.08	619.208	1.899	P1	8.05	11.254	6.946	90	82.95	82.6
11	0.08	618.19	1.903	P1	8.064	11.19	6.954	90	82.96	83.07
12	0.092	618.581	1.901	P1	8.03	11.267	6.949	90	82.92	82.58
13	0.092	618.612	1.901	P1	8.044	11.24	6.95	90	83.01	82.76
14	0.092	618.129	1.903	P1	8.044	11.221	6.954	90	82.91	83
15	0.092	617.886	1.904	P1	8.042	11.22	6.952	90	82.82	83.19
16	0.092	618.083	1.903	P1	8.056	11.204	6.952	90	82.9	83.11
17	0.092	617.923	1.903	P1	8.053	11.201	6.955	90	82.94	83.04
18	0.138	616.16	1.91	$P2_1$	8.657	8.441	8.442	90	87.41	90
19	0.161	616.034	1.909	P1	8.659	8.448	8.432	90	87.34	90
20	0.183	617.785	1.904	P1	8.061	11.177	6.96	90	82.81	83.23

Table S6 Comparison of the CL-20 crystal structures predicted by USPEX and experimental data.

	ρ(g/cm³)	a(Å)	b(Å)	c(Å)	α(°)	β(°)	γ(°)
**ε-CL-20 (exp)[1]**	2.04	8.84	12.56	13.38	90	106.9	90
**ε-CL-20 (exp)[2]**	2.04	8.86	12.59	13.40	90	106.92	90
**ε-CL-20 (USPEX+ReaxFF-nn)[3]**	2.04	8.98	12.40	13.42	90	105.90	90
**CL-20 (USPEX+DFT)**	2.04	8.85	12.57	13.39	90	105.45	90
**γ-CL-20 (exp)[1]**	1.91	13.21	8.16	14.89	90	109.17	90
**CL-20 (USPEX+DFT)**	1.98	13.28	8.23	14.82	90	109.17	90

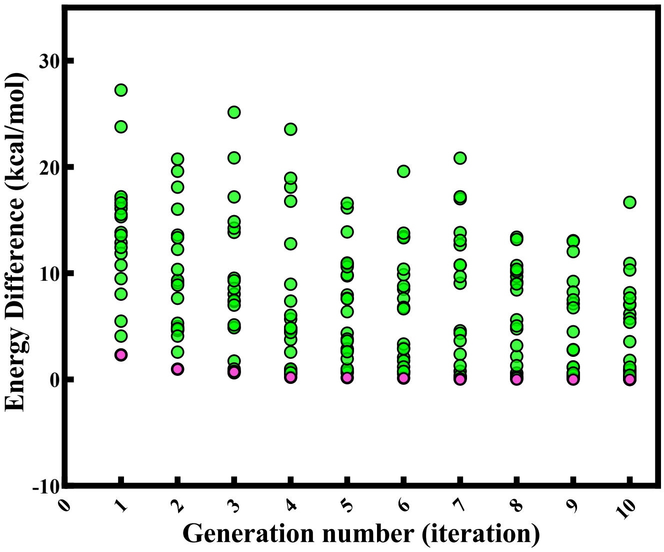

Figure S1. Relative energies of CL-20 molecular structures predicted by USPEX of all generations.

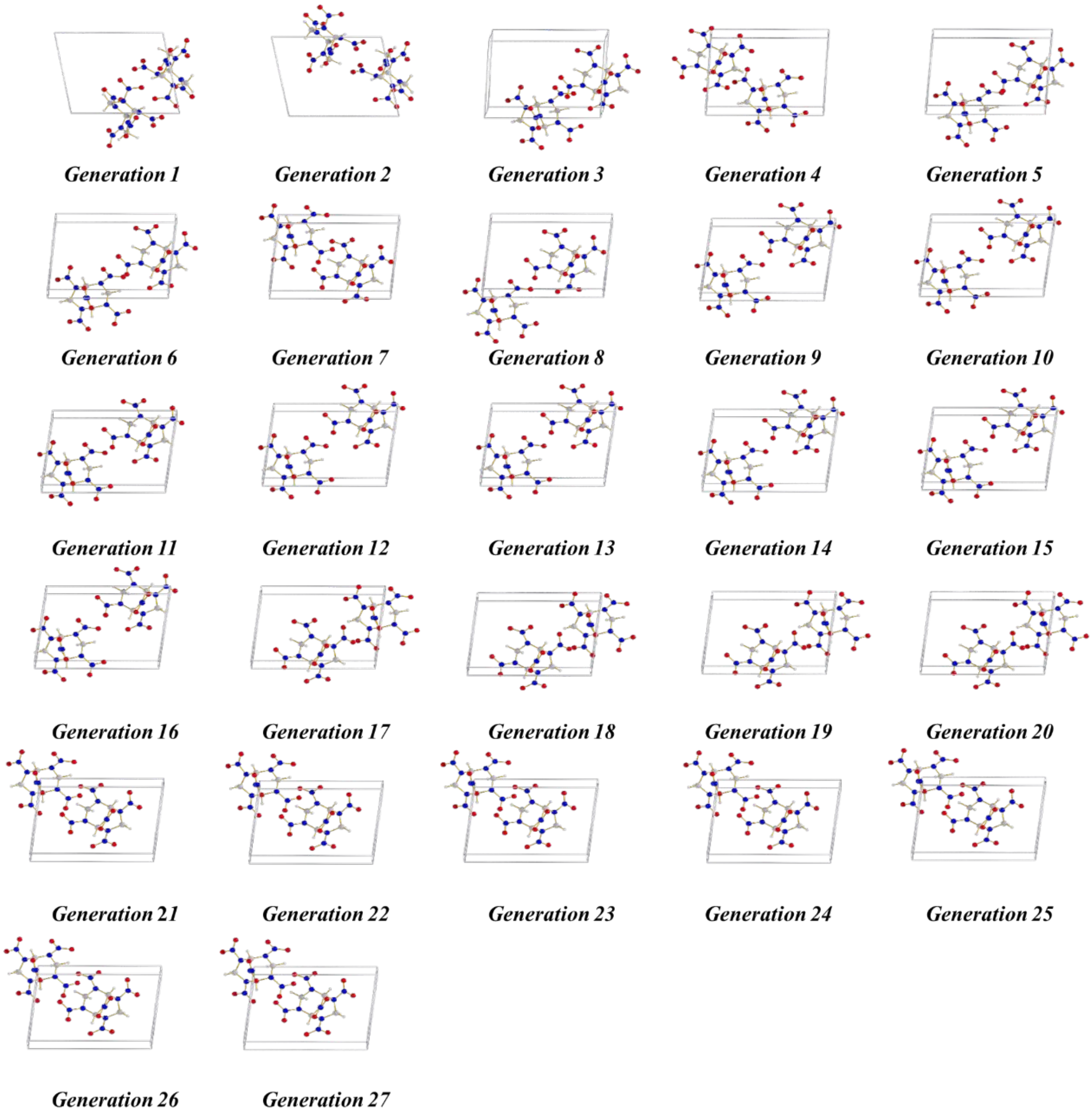

Figure S2. The predicted lowest energy structures from 27 generations by USPEX of BCHMX-ENO.

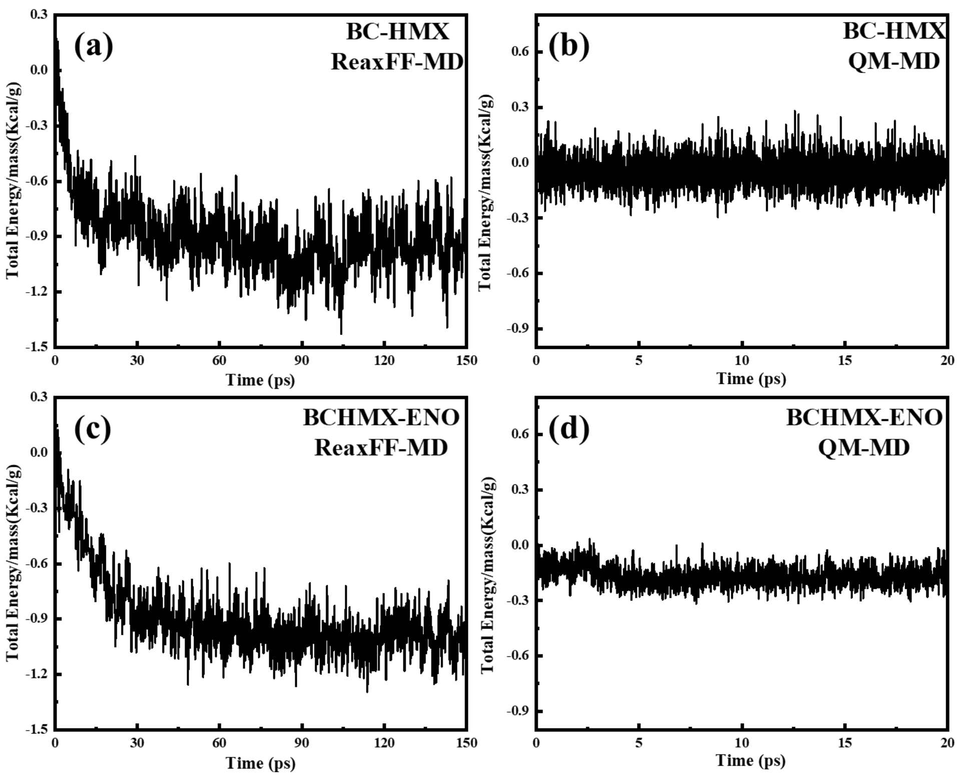

Figure S3. Time evolution of the total energy per unit mass in the cook-off simulation. The initial energy was set to zero as a reference: (a) RxMD for the first 150 ps, (b) QM-MD for the last 20 ps at T = 3000 K and V/V_0 = 0.75 for the BCHMX system, (c) RxMD for the first 150 ps and (d) QM-MD for the last 20 ps at T = 2600 K and V/V_0 = 0.65 for the BCHMX-ENO system.

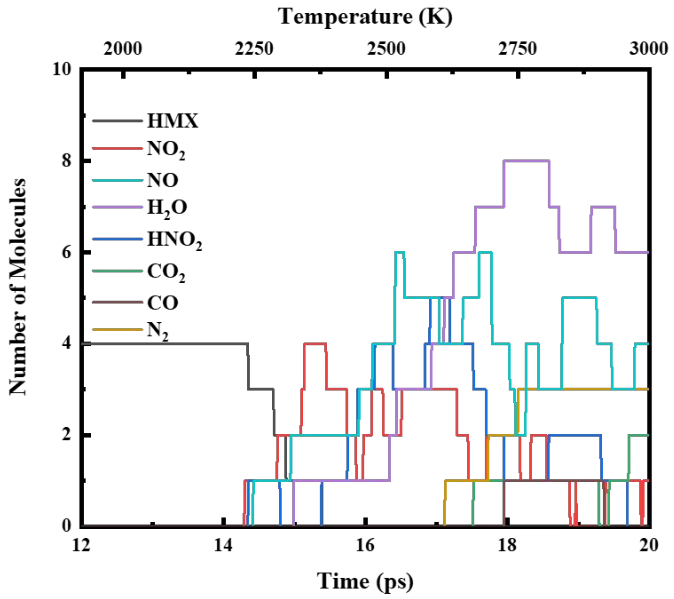

Figure S4. Species analysis for the decomposition of β-HMX heated from 300 to 3000 K over 20 ps

References

[1] ZQ, Wang.; HB Zhang.; Liu Y.; Hu SQ.; Hu LS.; Xu JJ. The effect of crystallization characteristics on polymorphic transformation laws and kinetics of CL-20. *Chin. J. Energ. Mater.* **2023**, *31*, 142-151.

[2] Russell, T. P.; Miller, P. J.; Piermarini, G. J.; Block, S. Pressure/Temperature Phase Diagram of Hexanitrohexaazaisowurtzitane. *J. Phys. Chem.* **1993**, *97*, 1993-1997.

[3] Ye, Z. H.; Guo, F.; Chai, C. G.; Wen, Y. S.; Zhang, Z. R.; Li, H. S.; Cui, S. X.; Zhang, G. Q.; Wang, X. C. Searching new cocrystal structures of CL-20 and HMX via evolutionary algorithm and machine learning potential. *J. Mater. Inf.* **2024**, *4*, 1-14.
